# Branch-specific gene discovery in cell differentiation using multi-omics graph attention

**DOI:** 10.1371/journal.pcbi.1013664

**Published:** 2025-11-03

**Authors:** Yihao Yin, Linzhi Zhuang, Yulei Wang, Yazhou Shi, Bengong Zhang

**Affiliations:** 1 School of Mathematics and Statistics, Wuhan Textile University, Wuhan, Hubei, China; 2 Research Center for Applied Mathematics and Interdisciplinary Studies, Wuhan Textile University, Wuhan, Hubei, China; Zhengzhou University of Light Industry, CHINA

## Abstract

Understanding gene regulation during cell differentiation requires effective integration of multi-omics single-cell data. In this study, we propose BranchKGN, a heterogeneous graph transformer-based framework for identifying branch-specific key genes along cell differentiation trajectories. By integrating scRNA-seq and scATAC-seq data into a unified gene representation, we infer differentiation trajectories using Slingshot and construct a heterogeneous graph capturing gene–cell relationships. Through attention-based graph learning, BranchKGN assigns gene importance scores within each cell, enabling the identification of genes consistently informative across branch point cells and their descendant lineages. These genes are then used to reconstruct gene regulatory networks and differentiation trajectories. Validation on three independent datasets demonstrates that the identified gene sets not only capture key regulators of cell fate bifurcation but also support accurate reconstruction of differentiation trajectories. Our results highlight the effectiveness of BranchKGN in dissecting gene regulation dynamics during cellular transitions and provide a valuable tool for multi-omics single-cell analysis.

## Author summary

Cell fate is an important biology process during biological development, tissue regeneration, and disease progression. However, the modes of cell differentiation and the patterns of gene expression changes during this process are still not so clear. To investigate these problems, we introduce BranchKGN, a framework based on graph attention mechanisms designed to identify branch-specific key genes along cell differentiation trajectories. By integrating scRNA-seq and scATAC-seq data and inferring differentiation trajectories using Slingshot, BranchKGN employs multi-head attention learning to score the importance of genes within each cell. And then branch-specific key genes at bifurcation points are identified. These genes are used to reconstruct gene regulatory networks and differentiation trajectories. We test BranchKGN on three independent datasets, can identify specific key gene sets at cell fate bifurcations. These key genes can accurately reconstruct the cell differentiation trajectories in turn. Our results highlight that BranchKGN is a powerful tool to dissect gene regulatory dynamics during cell transitions and analyze the multi-omics single-cell data.

## Introduction

Understanding the transitions between cellular states and fates is fundamental for deciphering the mechanisms of biological development, tissue regeneration, and disease progression [1–3]. During these processes, cells undergo dynamic changes in gene expression and chromatin accessibility, ultimately committing to distinct lineages and acquiring specialized functions[4–6]. Recent advances in single-cell technologies, particularly single-cell RNA sequencing (scRNA-seq) and single-cell assay for transposase-accessible chromatin using sequencing (scATAC-seq), provide high-resolution, multi-modal snapshots of individual cells [7,8]. These technologies offer unique opportunities to capture cell-to-cell heterogeneity and unravel the regulatory programs that drive fate specification.

Despite these advances, a key challenge remains: how to identify branch-specific regulatory genes that drive fate bifurcations in complex differentiation systems [9]. While traditional differential expression analyses can highlight global markers, they often fail to capture context-dependent regulators that selectively influence specific lineage choices at critical branch points [10,11].

Trajectory inference algorithms, such as Monocle [12,13], TIGON [14], and PAGA [15] have been widely adopted to reconstruct cellular developmental paths by projecting single-cell data onto low-dimensional manifolds and ordering cells along pseudotime or tree-like structures [16,17]. These methods effectively capture global lineage relationships and branch points but typically fall short in pinpointing the molecular regulators driving fate bifurcations. This is largely due to their focus on modeling cell-level dynamics, rather than explicitly quantifying gene-level contributions to fate decisions [18]. For instance, MuTrans [19] applies manifold learning to identify transitional states with maximum transcriptomic uncertainty, and SpliceJAC [20] estimates Jacobian matrices derived from RNA velocity to detect bifurcating states. While informative, such approaches primarily emphasize cell-level transitions, providing limited insight into gene-level drivers of differentiation.

Efforts have also been made to characterize gene regulatory mechanisms along developmental trajectories [21]. Notably, Topographer reconstructs a quantitative Waddington landscape from single-cell transcriptomic data, integrating macro-scale lineage topology, meso-scale gene regulatory networks, and micro-scale transcriptional burst kinetics [22]. It estimates transition probabilities and reveals increased transcriptional noise and gene–gene correlation heterogeneity near bifurcation points, offering valuable insights into the stochastic nature of fate decisions. However, Topographer relies solely on gene expression data and does not incorporate chromatin accessibility or multi-modal regulatory features, limiting its capacity to pinpoint causal, branch-specific regulators.

In parallel, deep learning has emerged as a powerful tool for modeling high-dimensional single-cell multi-omics data. Methods such as DeepMAPS [23], MarsGT [24] and GRNFormer [25] employ graph neural networks or transformer-based architectures to integrate gene expression and chromatin accessibility, jointly learning embeddings for genes and cells. These approaches enable the inference of gene regulatory networks (GRNs) [26] and can reveal cell-type-specific gene–gene interactions. However, most focus on global or pairwise regulatory patterns, and are not specifically designed to uncover the branch-specific regulators that determine bifurcation outcomes [27]. Moreover, identifying causal regulators in rare or transitional cell states remains particularly challenging due to data sparsity, noise, and the complexity of underlying regulatory programs.

To address these limitations, we introduce BranchKGN, a deep learning framework tailored to identify branch-specific key regulators by integrating scRNA-seq and scATAC-seq data. BranchKGN models gene regulatory landscapes as attributed graphs, where nodes represent genes with multi-omic features and edges encode potential regulatory associations. By leveraging a multi-head self-attention mechanism [28], the model computes gene-level scores that reflect their importance in distinguishing cell fates across divergent branches. BranchKGN then aligns cell clusters along inferred pseudotime trajectories and quantifies the dynamic rewiring of GRNs during differentiation. This framework enables the discovery of bifurcation-associated genes, offering mechanistic insights into lineage commitment and cell fate decisions. We demonstrate the effectiveness of BranchKGN on multiple real datasets, showing its ability to reveal interpretable, biologically relevant regulators that govern cell-type-specific transitions.

## Materials and methods

### BranchKGN framework

**Fig 1:**
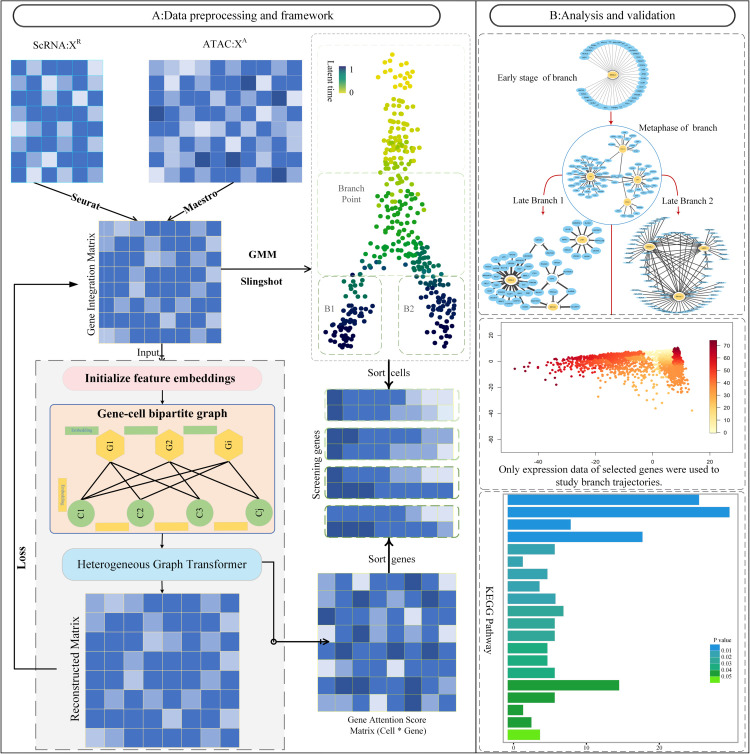
Overview of the BranchKGN framework. A: The framework consists of three main components: multi-omic data integration, trajectory inference, and gene-to-cell importance learning via attention-based graph modeling. B: The selected key genes are used to reconstruct differentiation trajectories and infer dynamic gene regulatory networks across pseudotime.

As shown in Fig 1, BranchKGN is a computational framework designed to identify branch-specific regulatory genes by integrating single-cell transcriptomic (scRNA-seq) and chromatin accessibility (scATAC-seq) data. Seurat is first applied to normalize scRNA-seq data, while MAESTRO computes Regulatory Potential (RP) scores from scATAC-seq [29,30]. The two modalities are integrated into a Gene Integration Matrix (GIM) using Seurat [31], capturing both gene expression and regulatory potential. The GIM is then reduced via PCA [32,33] and used for trajectory inference with Slingshot based on Gaussian Mixture Models (GMM) [34], which identifies branching points and partitions differentiation into pre-branching, branching, and post-branching phases.

In parallel, the GIM is transformed into a heterogeneous gene–cell graph, where nodes represent genes and cells, and edges encode expression, accessibility, or regulatory interactions [35]. A Heterogeneous Graph Transformer (HGT) is employed to learn embeddings and compute Gene Attention Scores (GAS) for each gene–cell pair, quantifying gene contributions to cell fate [36]. Branch cells identified from the trajectory are analyzed by ranking genes according to GAS, followed by filtering with a score threshold and expression probability (≥50% of branch cells). Genes passing these criteria are designated as branch-specific regulatory genes, which are further used to reconstruct differentiation trajectories and infer branch-specific gene regulatory networks (GRNs), see Fig 1B for details.

### Integration of scRNA-seq and scATAC-seq data

To capture transcriptional and chromatin accessibility signals at single-cell resolution, we integrated scRNA-seq and scATAC-seq data using the Seurat framework. Gene expression matrices from scRNA-seq were normalized with SCTransform [37], while scATAC-seq profiles were processed with TF-IDF [38] and converted into gene activity scores based on promoter and gene-body accessibility, reflecting regulatory potential [30] (detailed in S1 Text). The two modalities were aligned through canonical correlation analysis (CCA) to obtain a shared low-dimensional representation. This process yielded a harmonized Gene Integration Matrix that jointly encodes expression and accessibility features for matched cells, serving as the foundation for trajectory inference and graph-based modeling.

### Trajectory inference and branching phase definition

Based on the GIM, we first applied PCA to reduce the gene-level dimensionality, followed by cell clustering to identify major cell populations. Differentiation trajectories were reconstructed using Slingshot, which fits smooth lineages through clusters with Gaussian Mixture Models (GMMs) and detects bifurcation points [39,40]. Since this study focuses on bifurcating trajectories, only clusters located along bifurcations were considered for subsequent analysis. To assign clusters into pre-branching, branching, and post-branching phases, we applied the following criteria. For each cluster, we examined (i) the proportion of cells assigned to different lineages, (ii) the median pseudotime values across lineages, and (iii) the differences between lineage-specific pseudotimes. A cluster was classified as pre-branching if a substantial proportion of its cells were associated with multiple lineages (≥ρ) and if the median pseudotimes across lineages were small and highly similar (maximum difference <θ). Conversely, a cluster was classified as post-branching if the majority of its cells belonged to a single lineage (≥ρ) and if its median pseudotime was relatively large. Remaining clusters, which typically displayed intermediate pseudotime differences or heterogeneous lineage assignments without a dominant lineage, were classified as branching. In this study, we set the parameters *ρ*=2 and <θ=0.3. Given the varying number of cells and genes across different datasets, the resulting time spans also differ. Therefore, we recommend adjusting these parameters according to the specific characteristics of each dataset in practical computations.

### Construction of the gene–cell bipartite graph

From the integrated GIM, we constructed a heterogeneous bipartite graph with two node types: genes and cells. An undirected edge was added between a gene node and a cell node if the gene was expressed in that cell, with edge weights initially binarized (0 or 1) to indicate the presence or absence of an expression relationship, thereby encoding cell-specific transcriptional activity. Node representations were initialized using two independent linear neural networks that projected raw features of cells and genes into 256-dimensional embeddings. These embeddings were then used as inputs to the HGT for modeling gene–cell interactions.

### Heterogeneous graph transformer for gene–cell embedding

To capture the complex relationships between genes and cells, the gene–cell bipartite graph was processed by a multi-layer HGT in an unsupervised manner (see detail description in S1 Text) [23,41]. Each layer of the HGT consists of five components : (i) multi-head self-attention, where query, key, and value vectors are generated for each target–source pair according to node and relation types; (ii) message passing, propagating information between connected nodes; (iii) mutual attention, which quantifies the importance of gene–cell interactions; (iv) target aggregation, where weighted messages are integrated for each node; and (v) update and normalization, which refine the embeddings, as detailed in S1 Fig.

Specifically, each cell node j aggregates information from its connected gene nodes i∈N(j) via a multi-head attention mechanism. For each target cell j – source gene i pair, linear transformations generate query, key, and value vectors:

$$Q_{j}=W^{Q}\cdot h_{j}\;K_{i}=W^{K}\cdot h_{i}\;V_{i}=W^{V}\cdot h_{i}$$ (1)

where *h*_*j*_ and *h*_*i*_ are the embeddings from the previous layer, and *W*_*Q*_, *W*_*K*_, $W_{V}$ are learnable weight matrices specific to the node types and relation types [42,43]. The attention weight $e_{ij}^{h}$ of gene node i towards cell node j for head h is computed as:

$$e_{ij}^{h}=\frac{K_{i}^{T}\cdot Q_{j}}{\sqrt{d_{h}}},$$ (2)

where *d*_*h*_ is the dimension of the key and query vectors. Attention weights of all connected gene nodes are normalized to assess their relative importance to the target cell. The updated embedding of cell j is obtained by aggregating the weighted value vectors and integrating with its previous embedding.

Through multiple stacked layers, the model outputs cell embeddings, gene embeddings, and attention weights between gene–cell pairs. The embeddings of genes and cells are subsequently combined by inner product to reconstruct the original gene–cell matrix. Trained in an end-to-end, unsupervised manner by minimizing Kullback–Leibler (KL) divergence between the reconstructed gene–cell matrix $\hat{X}$ and the original Gene Integration Matrix X:

$${ℒ}_{KL}=\sum_{i,j}X_{ij}\log\frac{X_{ij}}{{\hat{X}}_{ij}},$$ (3)

Minimizing KL encourages the model to learn embeddings that accurately capture the original gene–cell relationships while preserving the underlying transcriptional and regulatory structure. The HGT model was implemented with a hidden layer dimension of 128, number of attention heads H = 16, number of graph neural network layers of 2 and trained for E = 100 epochs with the learning rate is initially set to 0.01 and then automatically adjusted during training and the optimizer is Adam. Additional hyperparameters and implementation details are provided in Table A in S1 Text.

After training, the attention weights from all heads and layers are combined and normalized to generate a GAS matrix; This matrix provides an interpretable measure of each gene’s contribution to specific cell populations and is subsequently used for identifying key genes specific to each branch. Seeing S1 Text for details.

$$\alpha_{ij}=\sqrt{\sum_{h=1}^{H}{\alpha_{ij}^{h}}^{2}}=\sqrt{\sum_{h=1}^{H}{\frac{exp(e_{ij}^{h})}{\sum_{k\in N(j)}exp(e_{kj}^{h})}}^{2}}.$$ (4)

### Identification of branch-specific regulatory genes

To identify branch-specific regulatory genes, we focused on cells corresponding to each pseudotemporal phase, with particular emphasis on branching-point cells. For each cell, genes were ranked according to their GAS derived from the trained HGT model. Genes with GAS above a predefined threshold [44–46] were selected as candidate regulators.

Across all cells within a given phase, we retained as branch-specific key genes those expressed in a majority of cells (≥50%). While this threshold may exclude rare transitional regulators with low expression, it offers a practical balance between reducing noise and capturing reproducible signals. These high-confidence regulators were subsequently used to reconstruct reduced differentiation trajectories that preserve the original branching topology and to infer branch-specific GRNs, thereby capturing dynamic transcriptional programs and lineage-restricted regulatory shifts during fate bifurcation.

### Trajectory reconstruction and gene regulatory network construction

To validate the identified branch-specific key genes, cellular differentiation trajectories were reconstructed using only these genes, following the same procedure as the full-gene analysis. The resulting trajectories exhibited high topological consistency with the original ones, confirming that the selected genes capture essential branching dynamics.

To investigate dynamic gene regulation during differentiation, Log2 Fold Change (Log2FC) was calculated for each gene between experimental and control groups, defined as $Log2FC=\log_{2}\;\;\left(\frac{x}{y}\right)$, where *x* and *y* denote the average expression levels of the gene in the experimental and control clusters, respectively. This metric quantifies differential expression and highlights transcriptional changes across pseudotime. Based on these expression dynamics and pseudotime information, branch-specific gene regulatory networks were inferred for each cluster using GENIE3 [47] or STREAM [48], providing insight into lineage-restricted regulatory programs during cell fate commitment.

### Dataset description

We used four single-cell multi-omics datasets to evaluate BranchKGN, show in Table 1. First, oRG-111 dataset profiles outer radial glia (oRG) cells in the developing human cerebral cortex using single-cell RNA sequencing [49]. oRG cells are a specialized neural stem cell population in the outer subventricular zone, exhibiting transcriptional programs distinct from ventricular radial glia (vRG) cells and playing essential roles in cortical expansion and gyrification. The dataset contains 393 cells and 25137 genes. From these, 111 oRG-associated genes, including TNC, PTPRZ1, FAM107A, HOPX, and LIFR, were curated as gold-standard labels. This dataset was used to benchmark BranchKGN against existing methods for key gene identification.

**Table 1:** Four datasets used in this work.

Dataset	Cell	Gene	Peak	Label	scRNA	scATAC
Human Outer Radial Glia	393	25137	0	Yes	${✓}^{*}$	$\times^{**}$
pbmc_unsorted_3k	2738	12837	66647	No	✓	✓
pbmc_unsorted_10k	10705	13958	83570	No	✓	✓
pbmc_granulocyte_sorted_3k	2308	13294	83624	No	✓	✓

${\;\;}^{*}✓$: Available ${\;}^{**}\times$: Not Available

PBMC datasets (*pbmc_granulocyte_sorted_3k*, *pbmc_unsorted_3k*, *pbmc_unsorted_10k*) [50] were generated by 10x Genomics from peripheral blood mononuclear cells (PBMCs) of healthy donors, using scRNA-seq and paired scATAC-seq (https://support.10xgenomics.com/single-cell-multiome-atac-gex/datasets). PBMCs originate from hematopoietic stem cells and differentiate into two major lineages: myeloid and lymphoid [51–53]. They mainly consist of lymphocytes (T cells, B cells, NK cells), monocytes, and dendritic cells, making them a widely used system for studying hematopoietic differentiation. The pbmc_granulocyte_sorted_3k dataset (∼3,000 cells) was sorted to exclude granulocytes, enabling focused analysis of non-granulocyte immune populations. In contrast, the pbmc_unsorted_3k and pbmc_unsorted_10k datasets (∼3,000 and ∼10,000 cells, respectively) retained all PBMC subtypes, thus offering broader cellular diversity and increased data complexity. Although lacking predefined gold-standard labels, the PBMC datasets enable evaluation of BranchKGN by assessing whether branch-specific key genes can reconstruct differentiation trajectories and gene regulatory networks that remain consistent with full-gene analyses and biologically plausible.

## Results

### Benchmarking on the oRG-111 labeled dataset

BranchKGN was primarily developed to identify branch-specific regulators driving lineage bifurcations. However, due to the lack of benchmark datasets with explicit branch-point labels, direct evaluation of this ability is currently not feasible. As a proxy, we evaluated the general capability of BranchKGN in detecting functionally relevant genes using the oRG-111 dataset, which provides experimentally validated oRG-specific gene labels. This setting allowed us to benchmark BranchKGN against existing key gene identification approaches, even though the dataset does not directly capture branch-point regulation.

We benchmarked BranchKGN against four representative approaches: three statistical methods summarized by Li et al. [54], DISP, MVP, and VST [8,55], as well as the widely used toolbox scGEAToolbox [56]. For each method, we selected genes under four ranking thresholds (Top 500, 1000, 2000, and 3000). In BranchKGN, the selection probability was fixed at 0.5, and only the ranking of genes was varied; the actual number of selected genes was substantially smaller than the given thresholds.

**Fig 2:**
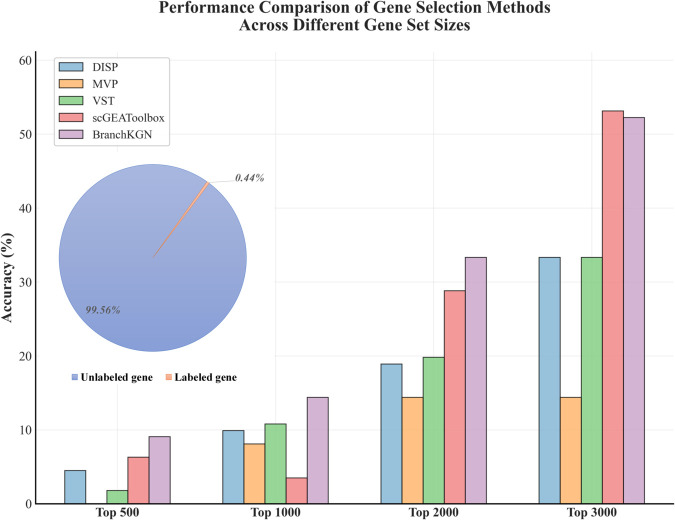
Accuracy of BranchKGN versus existing methods on the oRG-111 dataset. Bar plots show the percentage of correctly identified oRG-specific genes at four ranking thresholds (Top 500, 1,000, 2,000, 5,000) for DISP, MVP, VST, scGEAToolbox, and BranchKGN. A pie chart inset indicates the proportion of known oRG-associated genes in the dataset (111 genes, 0.44%), serving as the gold-standard reference.

The comparative results (Fig 2) demonstrate that BranchKGN consistently identifies oRG-specific genes with higher accuracy than the baseline methods, particularly at moderate ranking thresholds (Top 1000–2000). Notably, BranchKGN effectively prioritizes known oRG marker genes such as TNC, HOPX, and FAM107A, highlighting its strength in capturing biologically meaningful branch-specific signatures. However, we also observed that when the number of candidate genes was restricted to fewer than 1000, the accuracy of key gene identification decreased (<10%), indicating a limitation in sensitivity under stringent selection cutoffs. Although this dataset is not a strict benchmark for branch-point testing, these results demonstrate that BranchKGN exhibits a clear advantage in identifying functionally relevant key genes, laying the foundation for its application in branch-point scenarios.

### Identify branch-specific genes and differentiation trajectory inference

In order to find the branch-specific genes and infer the differentiation trajectory by BranchKGN, the “*pbmc_unsorted_3k* dataset” is used as a case study to illustrate branch-specific gene expression patterns (the results of other two datasets can be found in S4 Fig, S5 Fig and S6 Fig). Detailed methodological descriptions can be found in S1 Text.

**Fig 3:**
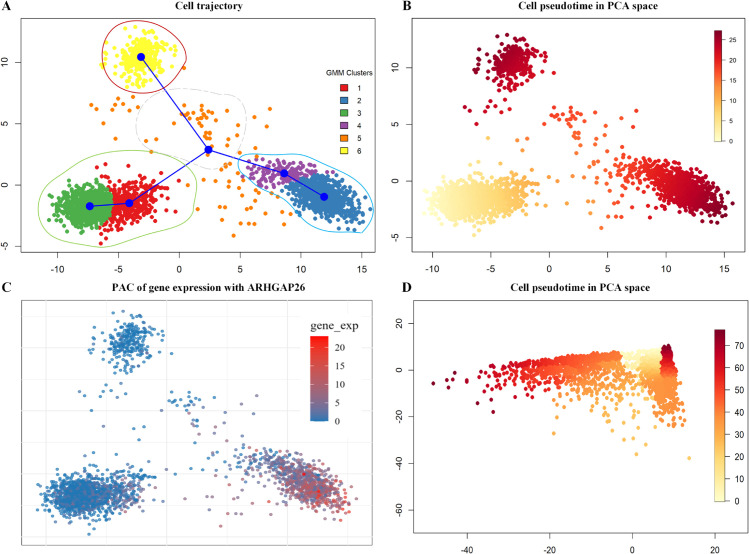
Pseudotemporal trajectory analysis and gene expression profiling for the *pbmc_unsorted_3k* dataset. A: Slingshot-inferred trajectory overlaid on cluster assignments, with six original clusters consolidated into four representative groups. B: Pseudotemporal ordering of all genes. C: Expression profile of the gene ARHGAP26 across clusters along the differentiation trajectory. D: Reconstructed trajectory using only the top 312 key genes.

By using MAESTRO, we integrated the gene expression matrix and the chromatin-derived gene activity scores into a shared low-dimensional space. PCA was then applied for dimensionality reduction, followed by GMM clustering, which identified six cell populations consistent with known biological annotations (Fig 3). Based on these clusters, Slingshot inferred cellular trajectories and revealed a clear bifurcation structure, allowing segmentation of the differentiation process into three phases: pre-branching (cluster 6), mid-branching (cluster 5), and post-branching (clusters 1/3 vs. clusters 2/4) (Fig 3A and 3B). To validate this classification, we further applied TFvelo [57], an RNA velocity-based trajectory inference method. The results (S3 Fig) confirmed that the overall divergence trajectories and key branching points were consistent with our initial reconstruction.

To illustrate branch-specific gene expression dynamics, we examined ARHGAP26 across the identified clusters. As shown in Fig 3C, ARHGAP26 is highly expressed in clusters 2 and 4, while remaining low in clusters 1, 3, 5, and 6. Its temporal regulation along the pseudotime trajectory is evident: ARHGAP26 is upregulated during the mid-branching phase (cluster 5) and maintained in one post-branch lineage (cluster 6), but downregulated in the alternative branch. This highlights its branch-specific expression during the bifurcation process. To further demonstrate the generality of our method, S2 Fig presents expression patterns of four additional top-ranked genes, randomly selected from the highest-scoring candidates. These genes consistently show branch-specific patterns across different lineages, reinforcing the robustness and biological relevance of the key gene identification by BranchKGN.

Fig 3D shows the reconstructed trajectory using the top 312 key genes from the *pbmc_unsorted_3k* dataset. To assess reconstruction accuracy, we compared the pseudotime of all cells with that obtained using only branch-cluster reconstruction and calculated the Pearson correlation coefficient, which was 0.715. This significant positive correlation indicates that the reconstructed trajectory largely preserves the ordering of cells along the differentiation pathway.

### Verification of branch-specific genes and construction of gene regulatory networks

Using HGT method in Sect 3.1, we identified 312 genes with relatively high expression levels in mid-differentiation cells from a total of 12,837 genes. To evaluate whether these genes represent key regulators, we analyzed their expression dynamics across four distinct cell clusters and calculated average expression scores for each cluster.

**Fig 4:**
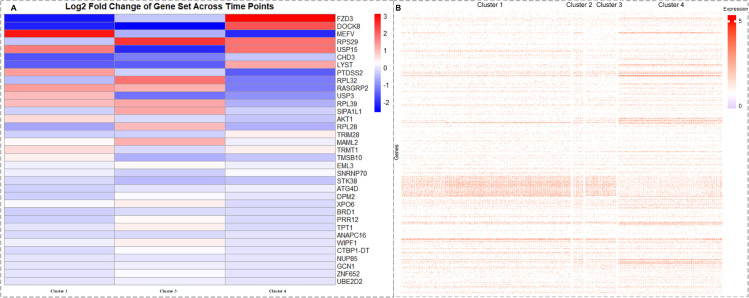
The expression variation of transfer genes. A: Log2FC variation of gene sets at different branches. B: Heatmap of the expression of selected genes in differentiated clusters.

As shown in Fig 3A, the trajectory can be divided into four sequential stages: pre-branching (cluster 1), mid-differentiation (cluster 2), and two post-branching lineages (clusters 3 and 4). To capture biologically meaningful transcriptional transitions, differential expression analysis was conducted only between adjacent stages along the trajectory, using cluster 2 as the reference cluster for comparisons with the other three clusters to assess statistically significant differences in gene expression, as shown in Fig 4A. Specifically, we compared (i) cluster 1 vs. cluster 2, (ii) cluster 2 vs. cluster 3, and (iii) cluster 2 vs. cluster 4.

For each pairwise comparison, average expression levels of the 312 candidate genes were computed, and two-sample t-tests were performed to assess statistical differences. P-values were adjusted using the Benjamini–Hochberg procedure to control the false discovery rate. As summarized in Table 2, a modest but non-significant increase was observed from cluster 1 to cluster 2 (Log2FC = 0.50, FDR = 0.114), whereas cluster 2 showed significant downregulation relative to clusters 3 and 4 (Log2FC = –0.99 and –1.32; FDR = 0.010 and 0.023, respectively). Non-adjacent comparisons (e.g., cluster 1 vs. clusters 3 or 4) were excluded, as they do not reflect direct sequential differentiation and may be biologically misleading.

**Table 2:** P-Value and FDR-adjusted p-value are used to judge the significance of Log2FC.

Comparable group	Log2FC value	P-value	FDR-adjusted p-value	Significance judgement
Cluster 1 vs Cluster 2	0.50405	0.11425	0.11426	non-significant
Cluster 3 vs Cluster 2	-0.99394	0.00202	0.01008	significant
Cluster 4 vs Cluster 2	-1.31797	0.00924	0.02310	significant

The pronounced reduction in gene expression in cluster 2 suggests transcriptional repression during mid-differentiation, with gradual reactivation in the later stages. Fig 4B presents a heatmap illustrating the expression profiles of the selected genes across the four clusters.

**Fig 5:**
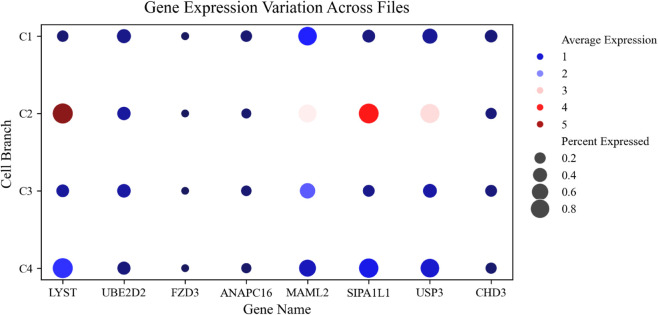
Regulation map of partial gene expression in different branches. This graph shows the changes in the average expression level and percentage of multiple genes in different cell branches (C1, C2, C3, C4). The dot color in the figure represents the average expression level of the gene, the darker the color, the higher the expression level, while the dot size indicates the percentage of the gene expressed in the cell, that is, how many cells express the gene.

**Fig 6:**
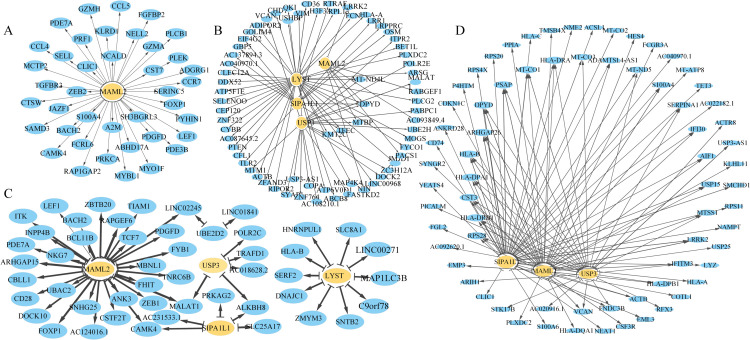
GRNS at different branch points. A: Pre-branching network centered on MAML2, emphasizing its regulatory interactions with various genes. B: Branching point network focused on USP1. C: Post-branching phase 1 network with MAML2. D: Post-branching phase 2 network featuring MAML2, USP1, and SIPA1L1. In each panel, nodes represent genes, where key genes are highlighted at a larger size, and the thickness of edges reflects the strength of interactions. These networks reveal the dynamic rewiring of regulatory interactions at different stages of cell differentiation.

To construct the gene regulatory networks, eight genes are randomly selected from the scoring table: ANAPC16, CHD3, FZD3, LYST, MAML2, SIPA1L1, UBE2D2, and USP3. First, the average expression of these eight genes in different branches is drawn in Fig 5, and it could be seen that the expression of LYST, MAML2, SIPA1L1, UBE2D2, USP3 was significantly different. The gene regulatory network is then constructed for different branches, as shown in Fig 6.

Using Genie3 to filter the “importance” value in each branch, it can be seen that MAML2 is expressed in all four branches and dominates before differentiation in Fig 6. During differentiation, LYST, SIPA1L1 and USP3 began to express. In addition, MAML2 and SIPA1L1, LYST and USP3 were expressed in post-branch 1 and post-branch 2, respectively, it shows differences in gene expression. As can be seen from the Fig 5, the heterogeneity of gene expression in different cell branches, for example, the expression levels and percentages of genes LYST, MAML2, SIPAIL1 and USP3 in C2 branch are significantly higher than in other branches.This may indicate the functional importance or regulatory differences of these genes in specific cell branches.

### Biological function analysis of branch-specific genes

In this section, the biological function analysis will be made for the branch-specific genes. It resorts to enrichment analysis which is a technique widely used in the field of bioinformatics to identify key genes and related pathways from large amounts of genetic data. It consists of several systematic steps as following:

1. Preliminary collection and collation of gene data in the selected library to ensure the completeness and accuracy of the data.
2. ClusterProfiler R package was used to realize KEGG [58] and GO [59] pathway analysis.
3. Evaluate the number of genes and their corresponding p-values for each biological process.

**Fig 7:**
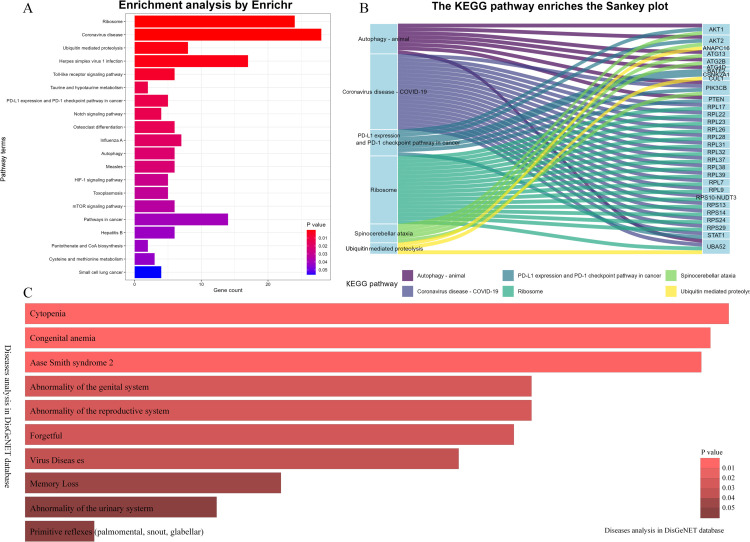
Enrichment analysis by KEGG,GO and DisGeNET. A: KEGG enrichment analysis showing significant pathways. B: KEGG pathway enriches the sankey plot. C: List of diseases associated with the analyzed gene set, ranked by their prevalence or significance.

To investigate the biological function of the branch-specific genes, we leveraged DisGeNET [60], a comprehensive database integrating gene–disease associations. Enriched biological processes were ranked based on their p-values, and the top 20 terms were visualized using bar plots (Fig 7). Statistically significant terms (*p* < 0.05) were highlighted with color-coded bars to facilitate interpretation. These results offer key insights and potential entry points for further investigation into the molecular mechanisms underlying the identified gene sets.

In addition to the pbmc_unsorted_3k dataset, we further applied BranchKGN to two additional PBMC datasets (pbmc_granulocyte_sorted_3k and pbmc_unsorted_10k). The results, shown in S4 Fig–S6 Fig, reveal consistent findings: trajectories reconstructed by BranchKGN preserve the branching topology, while the identified branch-specific regulators align with biologically relevant pathways. Notably, even for the larger 10k dataset, BranchKGN robustly recovered coherent differentiation trajectories and stage-specific GRNs, underscoring the scalability and reliability of our method.

## Discussion and conclusion

To deeply characterize gene expression dynamics during cell differentiation, we propose a graph attention network-based analysis framework, BranchKGN, designed to identify branch-specific key genes. By integrating scRNA-seq and scATAC-seq data across three real datasets, BranchKGN demonstrates robust performance in capturing cell fate transitions. Using HGT model, Slingshot algorithm and GMM clustering, we delineate the differentiation trajectory into four key stages. This staging not only spans the full temporal course of differentiation but also accurately captures critical transitions near branch points. Gene expression patterns specific to each stage are clearly resolved, highlighting the method’s ability to reveal regulatory dynamics at single-cell resolution. BranchKGN thus provides a valuable tool for dissecting cell state transitions and regulatory mechanisms during differentiation.

However, there is also potential limitation in the current study. BranchKGN currently incorporates only scRNA-seq and scATAC-seq data, without fully addressing inter-tissue heterogeneity, which may limit its generalizability across diverse biological contexts. Additionally, the trajectory inference approach is relatively simple, modeling only bifurcating structures and lacking resolution for more complex differentiation paths. Future work should focus on integrating spatial transcriptomics and broader multi-omics data, as well as enhancing trajectory modeling, to better capture the intricate regulatory networks underlying cell differentiation.

## Supporting information

S1 Text Details of scRNA-seq and scATAC-seq data integration, the principles and implementation of the Heterogeneous Graph Transformer, and the parameter settings (Table A). (DOCX)

S1 Fig Schematic overview of the Heterogeneous Graph Transformer (HGT). The framework takes as input a gene–cell bipartite graph with initial embeddings. Its core principle is to enable information exchange between nodes through graph edges, while employing an attention mechanism to dynamically weight the contributions of different neighbors. The model outputs low-dimensional embeddings for both cells and genes, together with attention scores that quantify the importance of each gene to each cell.
(TIFF)

S2 Fig Expression patterns of four representative top-ranked genes (LYST, DOCK8, MAML2 and UBE2D2) identified by BranchKGN. LYST and DOCK8 exhibit strong branch-specific expression in Branch 1, with little or no expression in Branch 2. MAML2 remains expressed both before and after branching and functions as a transcriptional co-activator in the Notch signaling pathway. UBE2D2 is expressed across both branches and encodes the ubiquitin-conjugating enzyme E2D2, a key regulator of protein degradation, cell cycle progression, and signal transduction. These results exemplify the branch-specific and biologically relevant expression patterns consistently captured by our method.
(TIFF)

S3 Fig The velocity flow graph obtained using Tfvelo. Cells in cluster 1 exhibit a coherent outward velocity toward cluster 3. Subsequently, cells in cluster 3 diverge into two distinct trajectories: one flowing toward clusters 5 and 6, and the other toward clusters 2 and 4. This dynamic pattern confirms the bifurcating differentiation process identified in our trajectory analysis.
(TIFF)

S4 Fig Results of the Pbmc_granulocyte_sorted_3k datasets. (A) Slingshot trajectory analysis with GMM clusters, illustrating the pseudotime progression of cells through different developmental stages. (B) Distribution of cell pseudotime in PCA space, color-coded by pseudotime (red = early stages, yellow = later stages). (C) Trajectory mapping using selected gene expression data, showing the transition of cells across principal components. (D) Pseudotime representation in PCA space, with a color gradient from light (early) to dark (late). (E) Bar plot of GO and KEGG enrichment analysis, where bar length represents gene count and color intensity reflects statistical significance (adjusted p-value). (F) GO analysis of differentially expressed genes, highlighting enriched biological processes, molecular functions, and cellular components.
(TIFF)

S5 Fig The result of analysis for *Pbmc_unsorted_10k* datasets. (A) Slingshot trajectory analysis with GMM clusters, depicting lineage bifurcation. (B) Distribution of cell pseudotime in PCA space. (C) Trajectory mapping using selected gene expression data, showing smooth transitions across principal components. (D) Pseudotime representation in PCA space, with a color gradient from light (early) to dark (late). (E, F) KEGG and GO enrichment analysis of branch-specific genes, presented as bar plots where bar length indicates gene count and color intensity reflects adjusted p-value significance.
(TIFF)

S6 Fig Gene regulatory networks of the *Pbmc_granulocyte_sorted_3k* and *Pbmc_unsorted_10k* datasets. (A) GRN visualization for the *Pbmc_granulocyte_sorted_3k* dataset. Core genes are highlighted in brown. A1: Pre-branching network dominated by ZFP69, ZNF624, ZBTB16, ZNF80A, and PRDM1. A2: Network at the branching point, with ZBTB16, TBX21, IKZF3, and PRDM1 as key regulators. A3: Branch 1 network primarily regulated by ZBTB16, with sub-networks involving ZBF707, ZNF30, and CASZ2. A4: Branch 2 network featuring a dual-layer structure driven by ZBTB16, PRDM1, IKZF3, NFATC2, and TBX2. (B) GRN visualization for *Pbmc_unsorted_10k* dataset (core genes in green). with key genes including BCL11B, FZD3, ITK, CASZ1, NFE2, RBM44, RSPH3, ZFP69, and ZNF707.
(TIFF)
