# Supplementary material for: Branch-specific gene discovery in cell differentiation using multi-omics graph attention
Source: PLoS Comput Biol. 2025 Nov 3;21(11):e1013664. doi: 10.1371/journal.pcbi.1013664 (PMC12594343; doi:10.1371/journal.pcbi.1013664)
Supplement: S1 Text — (DOCX) [file pcbi.1013664.s001.docx]

Supporting information for

Branch-Specific Gene Discovery in Cell Differentiation Using Multi-Omics Graph Attention

1. **Data preprocessing**

The analysis begins with the input of matched scRNA-seq derived raw count matrix and chromatin accessibility matrix . In the scRNA-seq data matrix, rows represent genes while columns represent cells. Conversely, the scATAC-seq data matrix is structured with regulatory regions (peaks) as rows and cells as columns. Any row or column containing less than 0.1% non-zero values in each data matrix was excluded from further analysis. Quality control measures were implemented using Seurat v3, including criteria such as total read counts and mitochondrial gene ratios.

The node ATAC features were quantified from the Regulatory Potential (RP) scores of scATAC-seq data using MAESTRO, which reflects the cumulative regulatory impact of scATAC-seq peaks surrounding a given gene in specific cells. Subsequently, a regulatory score matrix was constructed based on MAESTRO. In this matrix, represents the regulatory potential of peak relative to gene , determined by the genomic distance between peak and gene .

Here, represents the distance from the center of peak to the transcription start site (TSS) of gene , while denotes the half-decay distance parameter (set at 10 kb). Length(exon) indicates the length of the exon where peak is located. As shown in Equation (1), the regulatory potential weight of peak on gene is typically calculated as . For any given gene , if the distance from peak to the tss of gene exceeds 150kb, the resulting value becomes smaller than 0.0005, and the regulatory potential score is assigned as 0 for computational convenience.

In MAESTRO framework, when peak is located within the exon region of gene , is calculated as . For peaks residing in exon regions, is set to 0, making equal to 1 according to the formula, with subsequent normalization by the total exon length of gene . However, if peak is located in the promoter or exon regions of any neighboring genes, is set to 0 (effectively excluding that peak). This design stems from our observation in extensive ATAC-seq data that highly expressed genes frequently show peaks in exon regions due to binding by transcriptional machinery like Pol II. To better align the model with gene expression patterns, MAESTRO incorporates signals from exon regions while normalizing for the bias that reads are more likely to map to longer exons. This normalization is achieved by dividing the total reads on exons by each gene total exon length.

The regulatory potential score of peak on gene in cell is ultimately computed as , where matrix B represents the binarized output, with indicating the presence (1) or absence (0) of peak in cell . By summing the regulatory potential scores of all peaks regulating the same gene, transform the original scATAC-seq matrix into a gene regulatory potential matrix through:

The resulting regulatory potential matrix shares the same dimensions (I genes × J cells) with the expression matrix , enabling integrated multi-omics analysis. This quantitative transformation preserves the spatial regulatory information while converting chromatin accessibility data into gene-centric regulatory scores compatible with transcriptomic data.

**2.Heterogeneous Graph Transformers**

**2.1 Graph definition**

The HGT framework is employed to learn graph embeddings that reveal gene-cell interactions. The framework takes a joint matrix X as input and outputs weighted attention scores representing the importance of genes to cells.

Formally, a heterogeneous graph is defined as , where represents the set of nodes, represents the set of edges, denotes the union of node types, and denotes the union of edge types. The node types and edge types are labeled through mapping functions: for node typing and for edge typing. For any pair of nodes connected by an edge , their meta-relationship is represented as .

**2.2 Vector linear mapping**

To generate initial embeddings for cells and genes, we employed two autoencoders with distinct architectures. The cell autoencoder progressively compresses each cell's gene expression data from the original I-dimensional space to an n-dimensional intermediate representation, and finally to an m-dimensional latent space (with m=512 and n=256). Similarly, the gene autoencoder reduces each gene's cellular expression profile from J-dimensions to n-dimensions before reaching the same m-dimensional space. Through this dimensionality reduction process, both cells and genes obtain unified 256-dimensional initial embedding representations. Notably, this target dimensionality is implemented as a tunable hyperparameter, with its optimal value being dataset-dependent.

The autoencoder outputs consist of reconstructed matrices and , maintaining identical dimensions to their respective input matrix. For both autoencoders, we adopted mean squared error (MSE) as the loss function to quantify the reconstruction error between input matrices and their reconstructed counterparts. Let denote the embeddings at the th layer where . In the th layer of HGT, is updated by combining and from the ()th layer of HGT. The embeddings of nodes and at the th layer of HGT are denoted as and respectively, while and represent the original value embeddings of and .

A multi-head attention mechanism is employed to evenly split and into heads. This multi-head attention mechanism allows different heads to focus on different information with distinct weights, with each head equipped with an independent attention mechanism, thereby enabling more efficient utilization of resources to generate output.

For the th head in the th layer of HGT, we use three linear mappings to update for each head (). These three linear mappings are:

In Equation **(1),** the function maps to the th query vector in the th layer of HGT, with the dimension changing from to , where is the dimension of and is the vector dimension of each head. Similarly, in Equations (2) and (3), and map to the th key vector and the th value vector respectively. The dimension changes from to , where is the dimension of and is the vector dimension of each head. Each node type has a unique linear mapping to maximally simulate distribution differences.

**2.3 Multi-head attention mechanism**

To estimate the importance of each neighboring node relative to node in the th attention head, we introduce an attention operator that computes . This operator maps each node pair to an output value representing importance to . The overall attention weight between and is obtained by concatenating attention weights across all heads. Here, Softmax denotes the normalized exponential function. The attention coefficients are computed as

The symbol represents a concatenation function, combining attention weights from all heads to produce the final attention coefficients between and . denotes the attention weight between and in the th head. is a transformation matrix capturing topological relationships. indicates matrix transposition. is a prior tensor representing the importance of each meta-relation , serving as adaptive scaling in the attention mechanism Parallel to mutual attention computation, we perform message passing from source to target nodes. Messages from to in head h are computed as:

,

where maps neighboring nodes to message vectors. is another transformation matrix integrating edge dependencies

The aggregation process combines information from all neighboring (source) nodes with different feature distributions into the target node v. During heterogeneous graph aggregation, the target node is the node requiring embedding updates, source nodes are all connected to via edges . At layer of HGT, we directly use attention vectors as weights to compute a weighted average of source node messages, yielding the updated vector :

The final step combines the trainable-weighted with to produce the new embedding for node v:

,

where is a trainable parameter, ReLU serves as the activation function,T his aggregation of information at the th HGT layer produces the final updated representation for node .

**2.4 Compute the attention score of the gene to the cell**

After the execution of the HGT process, the final attention fraction $a_{ij}$ of gene to cell can be obtained in the last HGT layer:

.

It is obtained by summing up the square root of all attention heads. This process integrates information from different heads of attention to measure the relative importance or similarity between node and node . The final attention score represents the numerical display of the importance of the gene to the cell. We believe that the higher the attention score of a gene to a cell, the more important the expression characteristics and heterogeneity of the gene in a specific cell.

**2.5 HGT training on subgraphs**

To enhance the efficiency and performance of the HGT model on large-scale heterogeneous graphs, we employ an HGT sampling method specifically designed for subgraph selection and model training. Taking cells and genes as an example, assume that the graph contains I gene nodes and J cell nodes. The subgraphs sampled should collectively cover (set as 30%) of the gene and cell nodes to ensure the effectiveness of model training. Consequently, the sampler constructs g subgraphs (defaulting to 50) from the original heterogeneous graph G, which are then fed into the HGT model in batches for training, leveraging multiple GPUs to accelerate computation. Each subgraph should encompass gene nodes and cell nodes.

Focusing on cell node j as the target node v, with its neighbor acting as the source node corresponding to gene i, we calculate the probability on edge as follows:

.

Here, represents the expression value of gene i in the integrated matrix X. For each target node v, we randomly select adjacent gene nodes based on the sampling probability .

The HGT hyperparameters, such as and , are trained and updated sequentially across subgraphs 1 to 50 within each epoch. Subgraph training is conducted in an unsupervised manner using a Graph Autoencoder (GAE). The HGT serves as the encoder layer, while the inner product of embeddings acts as the decoder layer. The loss function for the GAE is defined as the Kullback-Leibler (KL) divergence between the reconstructed matrix and the original matrix :

.

Subgraph training ceases when either the loss value reaches a predefined threshold or the training reaches a specified number of epochs, whichever occurs first.

**Table A. Detailed hyperparameter table of Heterogeneous Graph Transformer**

| Parameter Name | Type | Default | Help Information |
| --- | --- | --- | --- |
| n_layers | int | 2 | Number of GNN layers |
| sample_depth | int | 4 | How many numbers to sample the graph |
| sample_width | int | 8 | How many nodes to be sampled per layer per type |
| n_batch | int | 64 | Number of batch (sampled graphs) for each epoch |
| batch_size | int | 64 | Number of output nodes for training |
| reduction | str | Autoencoder | The method for feature extraction |
| in_dim | int | 256 | Number of hidden dimension (AE) |
| n_hid | int | 128 | Number of hidden dimension |
| n_heads | int | 16 | Number of attention head |
| dropout | float | 0 | Dropout ratio |
| lr | float | 0.01 | Learning rate |
| layer_type | str | 'Hgt' | The layer type for GAE |
| loss | str | 'Kl' | The loss for GAE |
