# Supplementary figures and images for: Branch-specific gene discovery in cell differentiation using multi-omics graph attention
Source: PLoS Comput Biol. 2025 Nov 3;21(11):e1013664. doi: 10.1371/journal.pcbi.1013664 (PMC12594343; doi:10.1371/journal.pcbi.1013664)

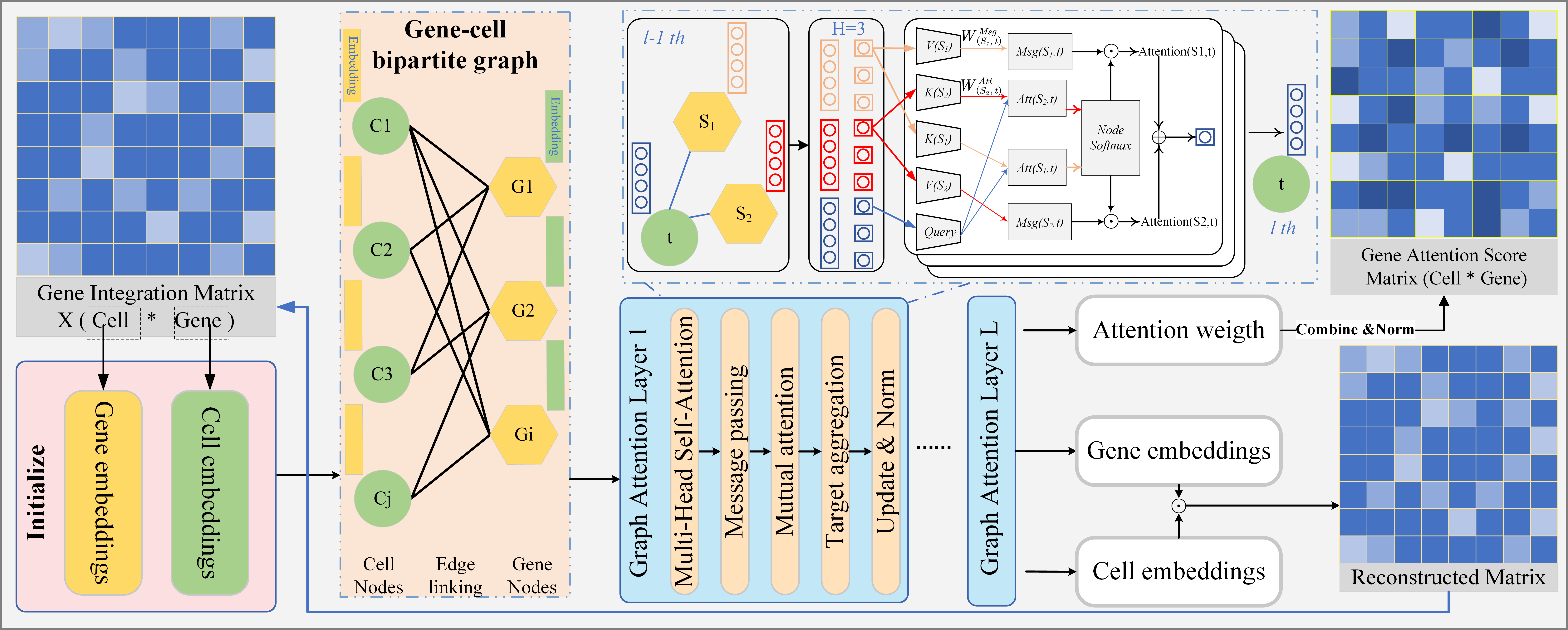

Supplement: S1 Fig — The framework takes as input a gene–cell bipartite graph with initial embeddings. Its core principle is to enable information exchange between nodes through graph edges, while employing an attention mechanism to dynamically weight the contributions of different neighbors. The model outputs low-dimensional embeddings for both cells and genes, together with attention scores that quantify the importance of each gene to each cell. (TIFF) [file pcbi.1013664.s002.tif]

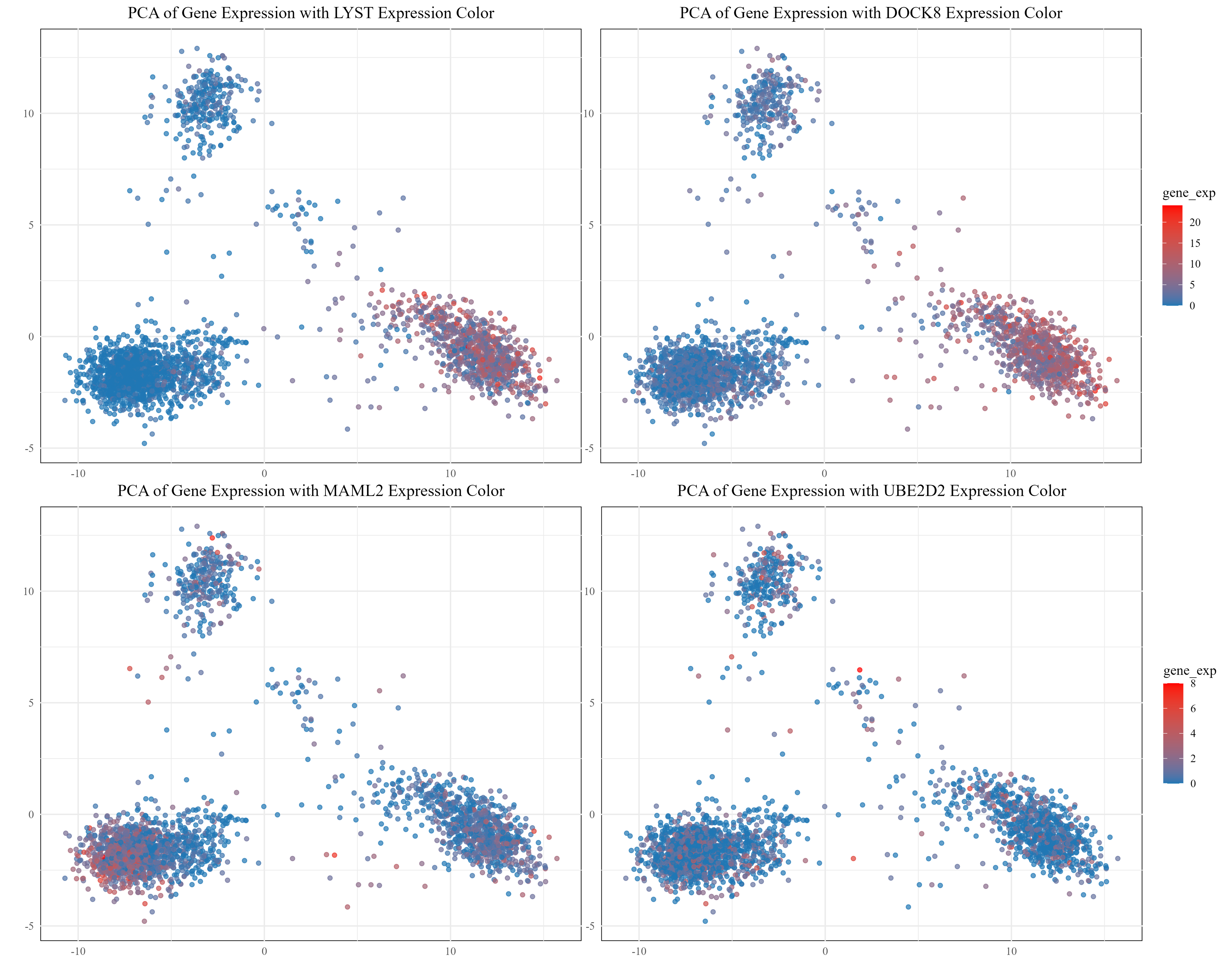

Supplement: S2 Fig — LYST and DOCK8 exhibit strong branch-specific expression in Branch 1, with little or no expression in Branch 2. MAML2 remains expressed both before and after branching and functions as a transcriptional co-activator in the Notch signaling pathway. UBE2D2 is expressed across both branches and encodes the ubiquitin-conjugating enzyme E2D2, a key regulator of protein degradation, cell cycle progression, and signal transduction. These results exemplify the branch-specific and biologically relevant expression patterns consistently captured by our method. (TIFF) [file pcbi.1013664.s003.tif]

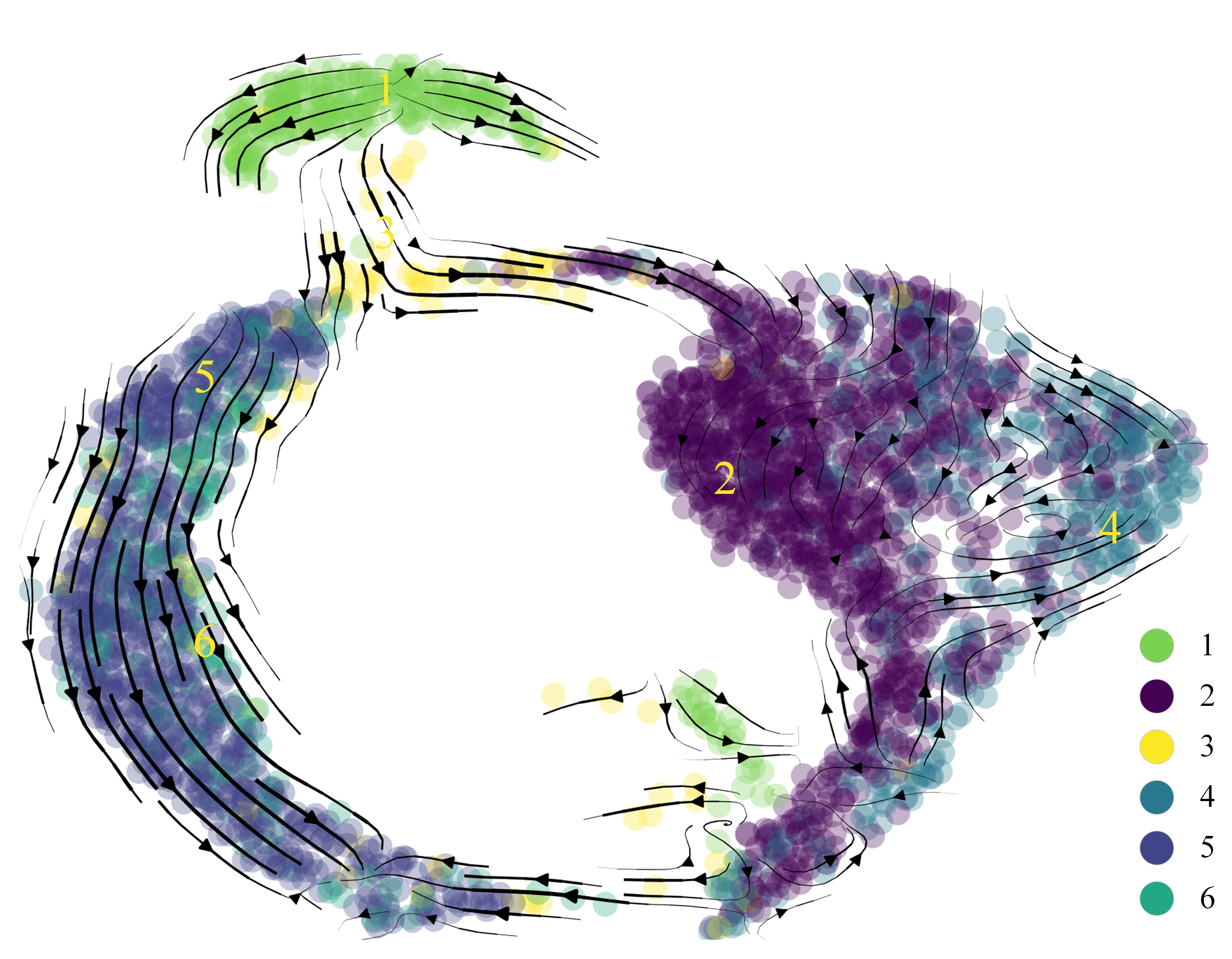

Supplement: S3 Fig — Cells in cluster 1 exhibit a coherent outward velocity toward cluster 3. Subsequently, cells in cluster 3 diverge into two distinct trajectories: one flowing toward clusters 5 and 6, and the other toward clusters 2 and 4. This dynamic pattern confirms the bifurcating differentiation process identified in our trajectory analysis. (TIFF) [file pcbi.1013664.s004.tif]
